# Improving plant salt tolerance through *Algoriphagus halophytocola* sp. nov., isolated from the halophyte *Salicornia europaea*

**DOI:** 10.3389/fmicb.2024.1466733

**Published:** 2024-10-21

**Authors:** Yuxin Peng, Dong Hyun Cho, Zalfa Humaira, Yu Lim Park, Ki Hyun Kim, Cha Young Kim, Jiyoung Lee

**Affiliations:** ^1^Korean Collection for Type Cultures (KCTC), Biological Resource Center, Korea Research Institute of Bioscience and Biotechnology, Jeongeup, Republic of Korea; ^2^Department of Agriculture and Life Sciences, Chonnam National University, Gwangju, Republic of Korea; ^3^Biosystems and Bioengineering, KRIBB School of Biotechnology Korea National University of Science and Technology (UST), Yuseong, Republic of Korea

**Keywords:** halophyte, novel species, plant growth-promoting bacteria, polyphasic taxonomy, salt stress, whole-genome sequence

## Abstract

*Salicornia europaea*, commonly known as glasswort, thrives in reclaimed land and coastal areas with high salinity, demonstrating remarkable adaptation to the arid conditions of such environments. Two aerobic, Gram-stain-negative, non-motile, rod-shaped bacterial strains, designated TR-M5^T^ and TR-M9, were isolated from the root of *Salicornia europaea* plants. These bacteria exhibit plant growth-promoting and salt tolerance-enhancing abilities, which have not been reported in other species of the genus. Both strains produce indole-3-acetic acid (IAA), a plant growth hormone, and synthesize proline, which functions as an osmoprotectant. Additionally, they possess gelatinase and cellulase activities. Cells grow in temperatures from 4 to 42°C (optimum 25°C), pH levels from 6.0 to 9.0 (optimum 7.0), and NaCl concentrations from 0 to 8.0% (optimum 6.0%). The average nucleotide identity and digital DNA–DNA hybridization values of strain TR-M5^T^ with the most closely related type strains for which whole genomes are publicly available were 74.05–77.78% and 18.6–23.1%, respectively. Phylogenetic analysis of their 16S rRNA gene sequences revealed that strains TR-M5^T^ and TR-M9 belong to the genus *Algoriphagus*. *A. locisalis* exhibited the highest similarity, sharing a sequence identity of 98.1%. The genomes of TR-M5^T^ and TR-M9 exhibit a G + C content of 43 mol%. This study specifically focuses on the identification and characterization of strain TR-M5^T^ as a novel species within the genus *Algoriphagus*, which we propose to name *Algoriphagus halophytocola* sp. nov., highlighting its potential role in enhancing plant growth and salt tolerance in saline environments. The type strain is TR-M5^T^ (KCTC 92720^T^ = GDMCC 1.3797^T^).

## 1 Introduction

Salt stress is a major abiotic factor that significantly impedes plant growth and productivity worldwide. High salinity levels in soil and water lead to osmotic stress, ion toxicity, and nutrient imbalance, adversely affecting plant health (Yang et al., 2023). These physiological and biochemical disruptions ultimately interfere with various aspects of plant development and metabolic functions (Rahman et al., 2016). To combat the adverse effects of salt stress, plants have evolved various adaptive mechanisms, often in collaboration with beneficial microorganisms.

Among these beneficial microorganisms, endophytes-microorganism that reside within plant tissues without causing harm—play a crucial role. Endophytes confer several benefits to their host plants, including enhanced growth, improved stress tolerance, and increased resistance to pathogens (Mitter et al., 2013; Omomowo and Babalola, 2019). Their ability to promote plant health under stress conditions makes them valuable assets in agricultural biotechnology.

Halophytes, or salt-tolerant plants, thrive in high-salinity environments and serve as a valuable reservoir for identifying salt-tolerant endophytes (Shurigin et al., 2020; Christakis et al., 2021; Gao et al., 2022). The endophytic bacteria and fungi associated with halophytes have co-evolved with their hosts to withstand harsh saline conditions, making them promising candidates for improving crop resilience in saline soils (Lee et al., 2017; Yuan et al., 2020; Gao et al., 2021). By harnessing the beneficial traits of these endophytes, it is possible to enhance the salt tolerance of agriculturally important crops.

Plant growth-promoting bacteria (PGPB) are a group of endophytes known to enhance plant growth and yield through various mechanisms. These include nitrogen fixation, phosphorus solubilization, hormone production, and the synthesis of osmoprotectants(Granada et al., 2018; Khabbaz et al., 2019). For instance, certain strains of *Pseudomonas* spp. produce siderophores that facilitate iron uptake, thereby improving plant growth and health under saline conditions (Nadeem et al., 2016; Mahajan et al., 2021). Similarly, *Bacillus* spp. produce extracellular polysaccharides that help maintain soil structure and moisture, promoting better water retention in plants subjected to salt stress (Amna et al., 2019; Kumar et al., 2021). The exploration of new PGPBs continues to be an important area of research for developing sustainable agricultural practices.

Recent studies have identified various endophytes from halophytes with unique mechanisms for enhancing plant salt tolerance. Some endophytic bacteria produce plant hormones such as indole-3-acetic acid (IAA) and abscisic acid (ABA), which regulate plant growth and stress responses (Sgroy et al., 2009; Zhao et al., 2016). Others synthesize osmoprotectants like proline, helping maintain cellular water balance and protein stability under salt stress (Altuntaş and Terzi, 2021). These findings highlight the potential of discovering novel endophytes with beneficial traits.

The genus *Algoriphagus*, a member of the family *Cyclobacteriaceae* within the phylum *Bacteroidota*, was first proposed by Bowman et al. (2003). The type species, *Algoriphagus ratkowskyi*, was isolated from sea ice and salt lake *cyanobacterial* mats (Bowman et al., 2003). As of now, there are 50 validly published species within this genus,^1^ which have been found in variety of environments, including seawater (Lee et al., 2012; Oh et al., 2012), freshwater and brackish lake (Liu et al., 2009; Muraguchi et al., 2016; Park et al., 2016), tidal flat sediments (Park et al., 2010), mangrove sediments(Yang et al., 2013), marine sediments (Han et al., 2017a; Han et al., 2017b), estuarine environments (Park et al., 2017a; Park et al., 2017b), and soil (Yoon et al., 2006; Young et al., 2009).

Members of the genus *Algoriphagus* exhibit a wide range of physiological and metabolic traits, reflecting their adaptability to diverse habitats. They are typically Gram-negative, aerobic, and exhibit gliding motility (Nedashkovskaya and Vancanneyt, 2015). Some species within this genus are thought to produce pigments like carotenoids, as suggested by the presence of biosynthetic gene clusters, which could play a role in promoting plant growth (Tao et al., 2006; Takatani et al., 2024). However, there have been no reported instances of *Algoriphagus* species functioning as plant growth-promoting bacteria (PGPB) or contributing to plant stress tolerance. This gap in knowledge suggests that *Algoriphagus* species may represent an underexplored resource for developing new strategies to enhance plant growth and resilience, especially under stress conditions such as high salinity.

This study focuses on the isolation and characterization of a novel endophytic bacterium, *Algoriphagus halophytocola* sp. nov., from the halophyte *Salicornia europaea*. We aim to investigate its potential as a PGPB with the ability to enhance plant growth and salt tolerance. By examining its production of IAA and proline, as well as its cellulase and gelatinase activities, we seek to demonstrate how this strain may contribute to nutrient availability and plant-microbe interactions in saline environments. Ultimately, this research explores the untapped potential of *Algoriphagus* species as PGPBs and their application in sustainable agriculture, particularly for improving crop productivity under conditions of soil salinity.

## 2 Materials and methods

### 2.1 Sample isolation and culture conditions

The roots of *Salicornia europaea* were first washed with tap water to remove surface soil and then weighed. One gram of root tissue was immersed in 10 ml of 1.3% sodium hypochlorite solution for 10 min with continuous agitation. Subsequently, the roots were rinsed thoroughly with 70% ethanol for 1 min, followed by several rinses with distilled water. The sterilized roots were ground in phosphate-buffered saline (1xPBS) at a ratio of 10 ml per gram of root weight using a blender (Jiang et al., 2020b). The resulting homogenate was spread onto marine agar (MA, Difco) plates and incubated at 25°C for 7 days. Individual colonies were isolated, cultured on fresh Tryptic Soy Agar (TSA, Difco) media, and purified for further analysis. These purified strains were preserved in 10% (w/v) skim milk at −80°C.

### 2.2 16S rRNA gene phylogeny

The 16S rRNA genes of strains TR-M5^T^ and TR-M9 were amplified through polymerase chain reaction (PCR) using the bacterial universal primers 27F and 1492R (Frank et al., 2008). The purified PCR products were sequenced by Macrogen Co. Ltd. (DaeJeon, Republic of Korea) employing primers 518F and 805R. The nearly complete 16S rRNA gene sequences (1,448 and 1,457 bp, respectively) were then submitted to the GenBank database.^2^ Phylogenetic neighbors were identified, and the sequence similarity of the 16S rRNA sequence was calculated using the EzTaxon.^3^

Sequence alignment was conducted using ClustalW within BioEdit (v7.0.5.3; Thompson et al., 1994). Phylogenetic trees were constructed utilizing neighbor-joining (NJ; Saitou and Nei, 1987), minimum-evolution (ME; Rzhetsky and Nei, 1992), and maximum-likelihood (ML) methods (Felsenstein, 1981) with MEGA version 7.0 (Kumar et al., 2016). Evolutionary distances between aligned sequences were calculated using the Kimura two-parameter model (Kimura, 1980), and the topologies of the phylogenetic trees were assessed using bootstrap methods with 1,000 replicates (Felsenstein, 1985).

### 2.3 BOX-PCR fingerprinting

Genomic fingerprinting, such as BOX-PCR, is a robust and effective tool for identifying and molecularly typing bacterial species (Koeuth et al., 1995). In the phylogenetic analysis of the 16S rRNA gene described above, it was observed that strains TR-M5^T^ and TR-M9 had identical sequences. To differentiate these two novel isolated strains, BOX-PCR fingerprinting analysis was employed to detect genetic variations among closely related species, specifically strains TR-M5^T^, TR-M9, and *Algoriphagus locisalis* KCTC 12310^T^. The genomic DNAs of these strains were utilized in the analysis. BOX-PCR was conducted using the BOX-A1R primer (5′-CTACGGCAAGGCGACGCTGACG-3′; Versalovic et al., 1994), following the cycling program described by Liu et al. (2021). Subsequently, the obtained PCR products were subjected to electrophoresis on 1% agarose gels to facilitate the comparison of the BOX-PCR fingerprints.

### 2.4 Overall genomic relatedness indices and phylogenomics

To comprehensively analyze the genomic characteristics of the isolated strains, whole-genome sequencing was performed using the PacBio Sequel System and Illumina sequencing platform. The sequence obtained were assembled using the Canu (v1.7) *de novo* assembler, followed by error correction with iPilon (v1.21) to rectify base errors, resolve mis-assemblies, and fill any gaps. Post-assembly, genome annotation was conducted using the NCBI Prokaryotic Genome Annotation Pipeline (PGAP; Tatusova et al., 2016).

To assess genomic relatedness, overall genomic relatedness indices (OGRI; Chun and Rainey, 2014) were employed, along with pairwise average nucleotide identities (ANI) values (Lee et al., 2016), calculated using OrthoANI version 0.93.1.^4^ Digital DNA–DNA hybridization (dDDH) values were determined using the Genome-to-Genome Distance Calculator 3.0 at http://ggdc.dsmz.de/ (Meier-Kolthoff et al., 2013). Additionally, average amino acid identity (AAI) values were obtained from the AAI calculator at http://enve-omics.ce.gatech.edu/aai/ (Rodriguez-R and Konstantinidis, 2014).

For a detailed phylogenomic analysis, whole-genome sequences of closely related type strains from the NCBI GenBank were included. The construction of a whole-genome-based phylogenetic tree was performed using the up-to-date bacterial 92 core gene set and pipeline (UBCG) set pipeline (Na et al., 2018). This involved identifying and extracting 92 single-copy core genes from complete genome sequences using HMMER. Gene prediction was performed with Prodigal, followed by individual alignments of the core genes using MAFFT. The concatenated alignments were then used to construct phylogenetic trees with FastTree. The robustness of tree branches was assessed using the Gene Support Index (GSI), which evaluates support from individual gene trees, ensuring high-resolution and reliable phylogenetic analysis.

### 2.5 Annotated genomic comparison of functional traits

To identify the closest genomic relatives of the new strains and to predict gene functions, we used the cluster of orthologous groups (COG) database (Tatusov et al., 2000) and KEGG annotations.^5^ Orthologous clusters among bacterial genomes were compared and visualized using Venn diagrams generated by OrthoVenn3 (Sun et al., 2023). Additionally, carbohydrate-active enzymes (CAZymes) were identified using dbCAN 3.0 (Zheng et al., 2023). This comparative genomic analysis insights into the functional traits and potential metabolic capabilities of the new strains.

### 2.6 Physiological characteristics

The growth parameters and physiological traits of strain TR-M5^T^ and TR-M9 were comprehensively evaluated using various techniques and conditions. The cellular morphology of strains TR-M5^T^ and TR-M9 was examined using scanning electron microscopy (FE-SEM, Regulus 8,100, Hitachi). Cells cultured on MA at 25°C for 3 days were initially fixed with 2.5% glutaraldehyde, followed by dehydration, critical point drying, and coating, following established procedures (Li et al., 2019). The prepared samples were then observed under the scanning electron microscope to visualize the cellular morphology.

To identify the optimal growth medium, strains TR-M5^T^ and TR-M9 were cultivated on various agar media, including Luria-Bertani agar (LBA, Difco), Reasoner’s 2A agar (R2A, MB cells), MA, TSA, and potato dextrose agar (PDA, Difco) for 7 days. The temperature range for growth was assessed by incubating the bacteria on MA at various temperatures (4, 10, 15, 20, 25, 30, 35, 40, 42, and 45°C) for 7 days.

The impact of NaCl concentration on bacterial growth was determined by culturing the bacteria in marine broth 2,216 excluding NaCl, and supplemented with varying concentrations of NaCl (ranging from 0 to 9% w/v, in 1.0% increments). Marine broth composition included 5.0 g peptone, 1.0 g yeast extract, 0.1 g FeC₆H₅O₇, 5.9 g MgCl₂, 3.24 g MgSO₄, 1.8 g CaCl₂, 0.55 g KCl, 0.16 g NaHCO₃, 0.08 g KBr, 0.034 g SrCl₂, 0.022 g H₃BO₃, 0.004 g Na₂SiO₃, 0.0024 g NaF, 0.0016 g NH₄NO₃, 0.008 g Na₂HPO₄ per liter of distilled water. Cultures were incubated at 25°C with agitation (150 rpm) for 7 days.

Optimal pH conditions were examined using marine broth adjusted to various pH values (ranging from 3.0 to 10.0, in 1.0 pH unit intervals) with a buffer system (Jiang et al., 2020a), incubated at 25°C with agitation (150 rpm) for 7 days. The tolerance of strain TR-M5^T^ and TR-M9 to different pH values and NaCl concentrations was evaluated by monitoring optical density measurements.

Catalase activity was evaluated by observing bubble production in 3% (v/v) hydrogen peroxide solution, while oxidase activity was assessed by testing the oxidation of 1.0% tetramethyl-p-phenylenediamine (bioMérieux). The Gram reaction was determined using the Gram Stain Solution kit (Difco) as per the manufacturer’s guidelines. Motility was assessed by observing diffusion growth on MA supplemented with 0.4% agar. The capability for anaerobic growth was tested by incubating on MA for 7 days at 25°C in an anaerobic chamber (Coy Scientific).

Additionally, a series of physiological and biochemical tests were performed using the API 20NE, API 50CH, and API ZYM kits (bioMérieux) according to the manufacturer’s instructions.

### 2.7 Chemotaxonomy characteristics

The chemotaxonomic characteristics of the strains were analyzed following established protocols outlined by Collins et al. (1980) and Minnikin et al. (1984). Both polar lipids and quinones were extracted from freeze-dried cells cultured in marine broth 2,216 at 25°C (19°C for *A. ratkowskyi*) with agitation at 150 rpm for 3 days.

For polar lipids analysis, samples were separated using two-dimensional thin-layer chromatography (TLC). Identification of the separated lipid was achieved using 0.2% ninhydrin (Sigma-Aldrich) and 4% phosphomolybdic acid spray. Respiratory quinone was extracted using 20 ml of chloroform/methanol (2:1, v/v) and analyzed by high-performance liquid chromatography (HPLC) with UV absorption at 275 nm.

After incubation for 72 h at 25°C (19°C for *A. ratkowskyi*) on MA, well-grown cell biomass was harvested for fatty acids analysis. The cellular fatty acids were prepared following the protocol of the Sherlock Microbial Identification System (MIDI; Sasser, 2006). This involved saponification, methylation, and extraction. The fatty acid profiles were then analyzed using gas chromatography (model 6890N, Agilent).

### 2.8 Plant materials and growth conditions

In this study, we used the *Arabidopsis thaliana* DR5::GUS transgenic plant (ecotype Columbia, Col-0 background; Ulmasov et al., 1997). Seeds were surface sterilized by soaking in 1.3% sodium hypochlorite for 10 min, followed by thoroughly rinsing five times with sterile water. The sterilized seeds were then resuspended in sterilized distilled water and kept in the dark at 4°C for 48 h to facilitate stratification.

Following stratification, the seeds were germinated on Murashige and Skoog (MS) medium for 7 days at 23 ± 2°C with a 16-h light/8-h dark cycle and a light intensity of 100 μmol m^−2^ s^−1^ for 7 days. Seven-day-old seedlings were subsequently transferred to 1/2 MS medium supplemented with 0 and 150 mM NaCl.

For each condition, 6–8 seedlings were cultured per plate, arranged in rows at equal intervals, with three replicates. The control (CK) group consisted of seedlings grown on untreated medium, while the experimental groups were co-cultured with bacterial strains for 7 days. The MA agar block was placed on 1/2 MS medium, and 10 μl of bacterial suspension with an OD_600_ of 0.1 was dispensed onto the MA agar block. The Petri dishes were positioned vertically under the previously described incubation conditions. Three replicates were conducted for each group to ensure statistical validity.

### 2.9 Determination of chlorophyll content

Chlorophyll content was extracted and measured according to Lorenzen’s procedure (Lorenzen, 1967) using a microplate spectrophotometer (Thermo Fisher Scientific, Multiskan Sky, USA). Whole leaf tissue was extracted with 95% ethanol, and each plant treatment was replicated eight times. The absorbance of the resulting supernatant was recorded at 645 nm and 663 nm. The total chlorophyll content (mg/L) was calculated using the following formula:


$$C\;\left(\mathrm{mg}/L\right)=20.2\;\times \;D_{645}+8.02\;\times \;D_{663}$$

The experiments were performed with 3 replicates using 8 seedlings per replicate.

### 2.10 *β*-Glucuronidase (GUS) histochemical staining

GUS activity was evaluated through histochemical staining with X-Gluc (GoldBio, St. Louis, MO, USA) following the method described by Jefferson (Jefferson et al., 1987). Seven-day-old seedlings, treated with or without strain JBR3-16 for an additional 14 days, were incubated in GUS staining solution containing 1 mM X-Gluc at 37°C for 24 h. After incubation, the stained roots were cleared overnight in 70% ethanol and examined at 40x magnification with a Carl Zeiss microscope (Axio Imager.A2) for imaging.

### 2.11 Plant growth promoting traits

The production of IAA was evaluated by culturing strains TR-M5^T^ and TR-M9 in 10 ml of MA medium with 0.1% L-tryptophan and incubating at 25°C with agitation at 130 rpm for 5 days. After incubation, cells were centrifuged, and 500 μl of the supernatant was mixed with an equal volume of Salkowski reagent [0.5 M FeCl_3_: distilled water: concentrated H_2_SO_4_ = 1:50:30 (v/v/v); Bric et al., 1991]. This mixture was then incubated in the dark for 30 min, and the absorbance was measured at 530 nm using a UV–vis microplate spectrophotometer (Thermofisher, USA).

Proline production was determined based on the method by Bates et al. (1973). Strains TR-M5^T^ and TR-M9 were cultured in 10 ml marine broth for 3 days. After incubation, the cultures were centrifuged, and the supernatant was retained. Sulfosalicylic acid was added to the supernatant to reach a final concentration of 3%, mixed thoroughly, and centrifuged again. Then, 1 ml of the supernatant was mixed with glacial acetic acid and acid-ninhydrin (prepared by dissolving 1.25 g ninhydrin in 30 ml glacial acetic acid and 20 ml 6 M phosphoric acid, with agitation). The mixture was incubated at 90°C for 1 h, cooled, and mixed with toluene. The absorbance of the upper phase was measured at 520 nm.

Siderophore production by TR-M5^T^ and TR-M9 was determined by placing agar disks from the cultures on CAS agar and incubating them for 7 days (Schwyn and Neilands, 1987).

The activities of cellulase, amylase, protease, and gelatinase were evaluated by incubating the strains on their respective agar plates for 3 days. Cellulase activity was determined on MA supplemented with 1% carboxymethylcellulose (CMC) and stained with 0.1% Congo red (Demissie et al., 2024). Amylase activity was assessed on MA containing 1% soluble starch, with iodine staining used for visualization (Gomez-Villegas et al., 2021). Protease activity was measured on a medium as follows (/L): 2.5 g tryptone, 80 g skim milk powder, 1.25 g yeast extract, and 15 g agar (Tang et al., 2018). Gelatinase activity was evaluated on MA supplemented with 1% gelatin(Torres et al., 2018).

Phosphate solubilization ability was evaluated using the National Botanical Research Institute phosphate growth medium (NBRIP). After initial growth on MA agar for 2 days, culture discs were transferred to NBRIP medium for a 5-day incubation (Gupta et al., 1994).

## 3 Results

### 3.1 16S rRNA phylogenetic analysis

Comparative analysis of 16S rRNA gene sequences revealed that the type strains most closely related to strains TR-M5^T^ and TR-M9 predominantly belong to the genus *Algoriphagus*. Among them, *A. locisalis* MSS-170^T^ exhibited the highest similarity, sharing 98.1% sequence identity, followed by *A. winogradskyi* LMG 21969^T^ with 97.6%, and *A. yeomjeoni* DSM 23446^T^ with 97.5%. The type species, *A. ratkowskyi* DSM 22686^T^, showed a sequence identity of 96.5%.

**Figure 1:**
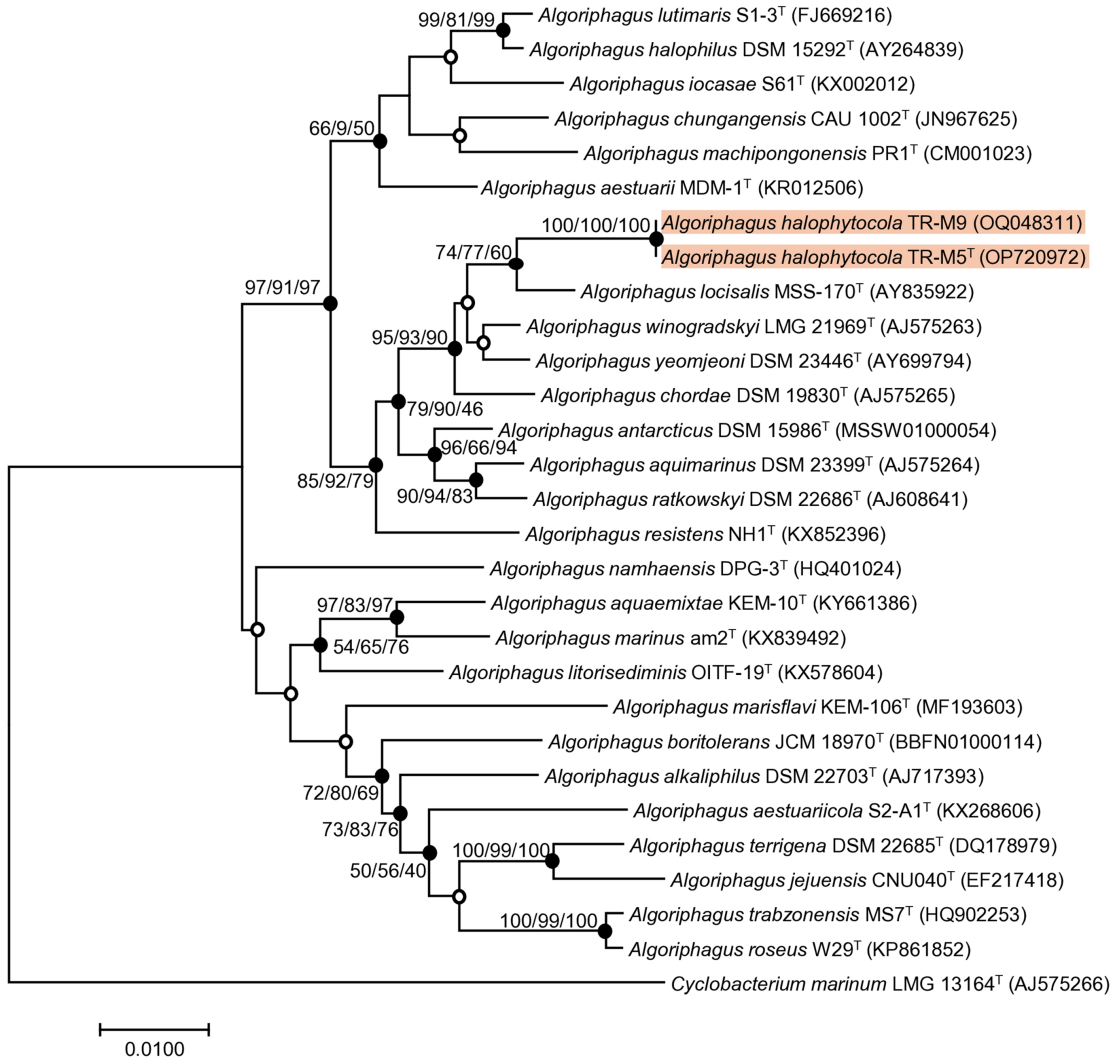
Phylogenetic tree of stain TR-M5^T^ and TR-M9. A phylogenetic tree was conducted using the neighbor-joining (NJ) algorithm based on the 16S rRNA sequence of strain TR-M5^T^, TR-M9 and closely related type strains. The numbers displayed on the branch nodes represent the percentage probability that the same branch exists, as determined using the NJ, maximum likelihood (ML), and minimum evolution (ME) algorithms, based on 1,000 bootstrap replicates (>50%). Filled circles on the nodes indicate that the relationships were also supported by either ML and ME algorithms, while open circles indicate nodes recovered by either the ML or ME algorithms. The scale bar corresponds to 0.010 changes per nucleotide position.

Further comparisons using the full-length 16S rRNA gene sequences via the EzBioCloud database confirmed these findings. Phylogenetic analysis based on 16S rRNA genes, employing NJ, ME, and ML methods, consistently placed strain TR-M5^T^ and TR-M9 within the *Algoriphagus* cluster. This analysis highlighted their distinct phylogenetic positions relative to other reference strains examined (Figure 1), showing close clustering with *A. locisalis* MSS-170^T^, *A. winogradskyi* LMG 21969^T^, *A. yeomjeoni* DSM 23446^T^, and *A. ratkowskyi* DSM 22686^T^.

In this study, we report the characterization of a novel bacterial strain, designated TR-M5^T^ which belongs to the genus Algoriphagus. For comparative analysis, four reference type strains were obtained from the Korean Collection for Type Cultures (KCTC) and Korean Agricultural Culture Collection (KACC). The reference strains used in this study include *A. locisalis* KCTC 12310^T^, *A. yeomjeoni* KCTC 12309^T^, *A. winogradskyi* KACC 12232^T^, and *A. ratkowskyi* KCTC 92714^T^.

### 3.2 BOX-PCR fingerprinting

The BOX-PCR fingerprinting analysis revealed distinct patterns for strains TR-M5^T^ and TR-M9, indicating that, although closely related, they are not clonal and represent distinct entities within their respective groups (Supplementay Figure S1). Furthermore, the fingerprints of TR-M5^T^ and TR-M9 differed from those of the most similar strain, *A. locisalis*, underscoring the genetic differences among the three strains.

### 3.3 Genome features and phylogenomic analysis

The genome characteristics of strains TR-M5^T^ and TR-M9 were thoroughly examined. Strain TR-M5^T^ possessed a genome length of 4,756,369 base pairs (bp), with an N50 value matching its total length. The G + C content of the genome was determined to be 43 mol%, with a sequencing depth of coverage of 199.3x. Conversely, the TR-M9 genome exhibited a length of 4,816,080 bp, also with an N50 value corresponding to its total length. The G + C content of the TR-M9 genome was slightly higher at 43 mol%, with a sequencing depth of coverage of 214.5x.

Comparative genomic analysis revealed OrthoANI values of 99.40, 77.78, 74.95, 75.16, and 74.05% for strains TR-M9, *A. locisalis*, *A. yeomjeoni*, *A. winogradskyi,* and *A. ratkowskyi*, respectively, in comparison to strain TR-M5^T^ (Supplementay Figure S2A). Similarly, their respective dDDH values are 94.8, 23.1, 19.3, 19.6, and 18.6% (Supplementay Figure S2B), while their AAI values are 99.30, 80.45, 78.69, 79.20, and 76.85% (Supplementay Figure S2C). These values fall below the acceptable classification thresholds of 95–96% for ANI, 70% for dDDH, and 95% for AAI (Luo et al., 2014; Riesco and Trujillo, 2024).

In terms of gene content, the TR-M5^T^ genome harbored 3,892 protein-coding sequences (CDSs) and 53 RNA genes. Among these RNA genes, three complete sets of rRNA genes (5S, 16S, 23S), 41 tRNA genes, and three non-coding RNA genes (ncRNAs) were identified. The completeness of the genome is 97.98% with a contamination level of 1.91%. Similarly, the TR-M9 genome comprised 4,009 protein-coding sequences (CDSs) and 52 RNA genes, including three complete sets of rRNA genes, 40 tRNA genes, and three ncRNA genes (Table 1) The completeness of the genome is 97.98% with a contamination level of 1.88%.

**Table 1:** Genomic characteristics of strains TR-M5^T^ and TR-M9.

Genomic characteristics	TR-M5^T^	TR-M9
GenBank accession number	CP110226	CP115160
Genome size	4,756,369	4,816,080
Contig 1	1	1
N50	4,756,369	4,816,080
L50	1	1
DNA G + C content	43 mol%	43 mol%
Genome Coverage	199.3	214.5
Gene (total)	3,961	4,078
CDSs (with protein)	3,892	4,009
Gene (RNA total)	53	52
tRNAs	41	40
rRNAs (5S, 16S, 23S)	3,3,3	3,3,3
ncRNA	3	3

Based on the eggNOG assignments, TR-M5^T^ had 3,781 genes functionally categorized into four categories (Supplementay Table S1). The majority of these genes belonged to the “Poorly characterized” category, accounting for a total of 45.83%, which included genes with unknown function (39.22%) and genes with general function prediction only (6.61%). The next most prevalent category was “Metabolism,” accounting for 25.97% of the total genes. Within this category, more than 5% of the gene clusters were related to amino acid transport and metabolism (6.27%), as well as inorganic ion transport and metabolism (5.18%). The remaining two categories were “cellular processes and signaling” (16.80%, including cell wall/membrane/envelope biogenesis at 6.61%) and “information storage and processing” (11.41%, including transcripts at 5.20%). TR-M9 had 3,833 genes with functional information to TR-M5^T^.

**Figure 2:**
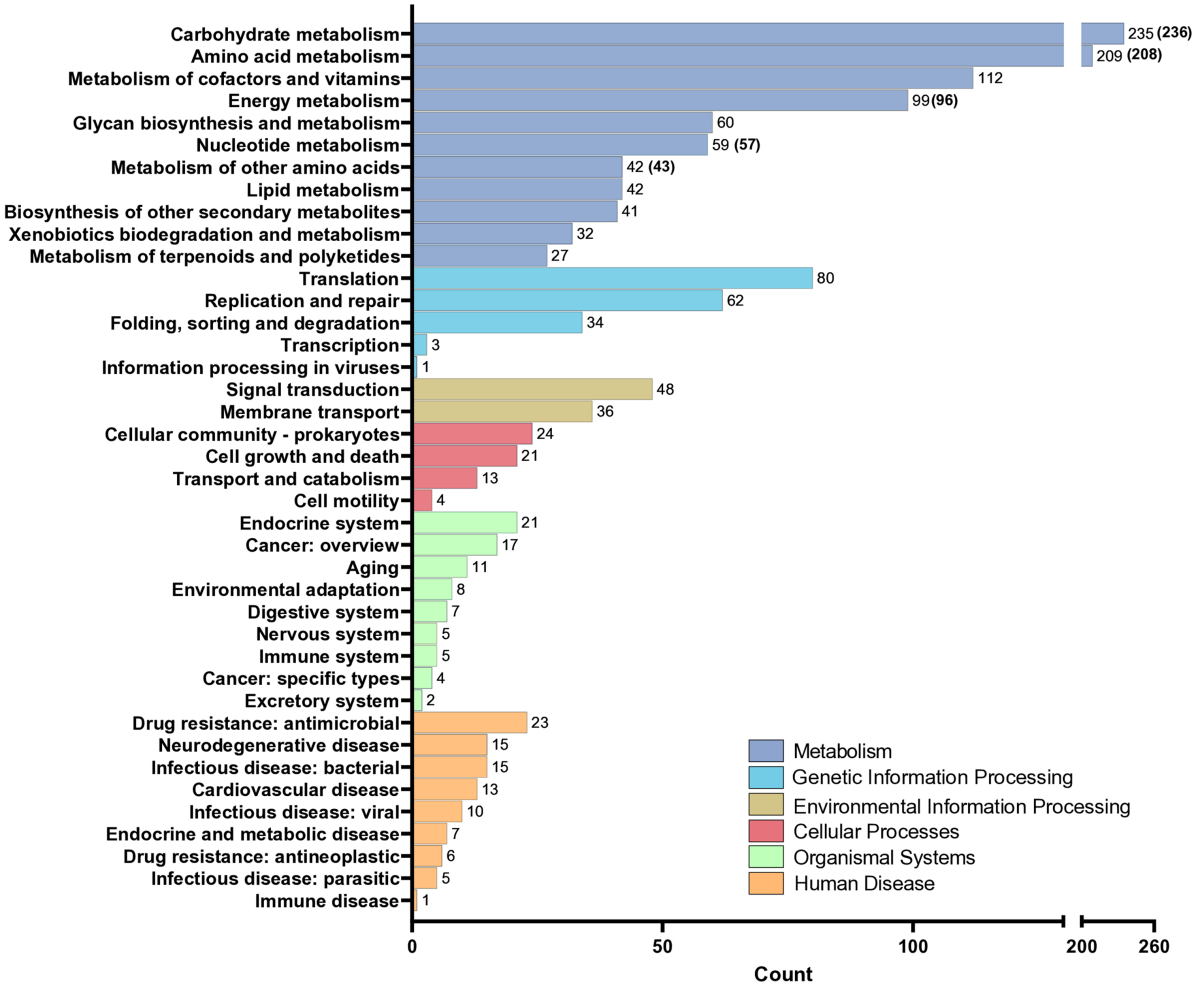
KEGG analysis of the genomes of strains TR-M5^T^ and TR-M9. KEGG analysis of the genomes of strain TR-M5^T^ and TR-M9 (values are shown in parentheses). Genes were classified into six main categories, with carbohydrate metabolism identified as the most abundant functional group.

The KEGG annotation process allowed for the identification of 38.1% of genes in the core genome, with a total of 48 complete pathway modules being identified. Within the core genome, a total of 1,459 enzymes were discovered (Figure 2). Notably, the category of metabolism was the largest in terms of gene representation. Specifically, carbohydrate metabolism stood out with its significant association with 235 genes for TR-M5^T^ (236 genes for TR-M9), followed closely by amino acid metabolism, encompassing 209 genes for TR-M5^T^ (208 genes for TR-M9).

**Figure 3:**
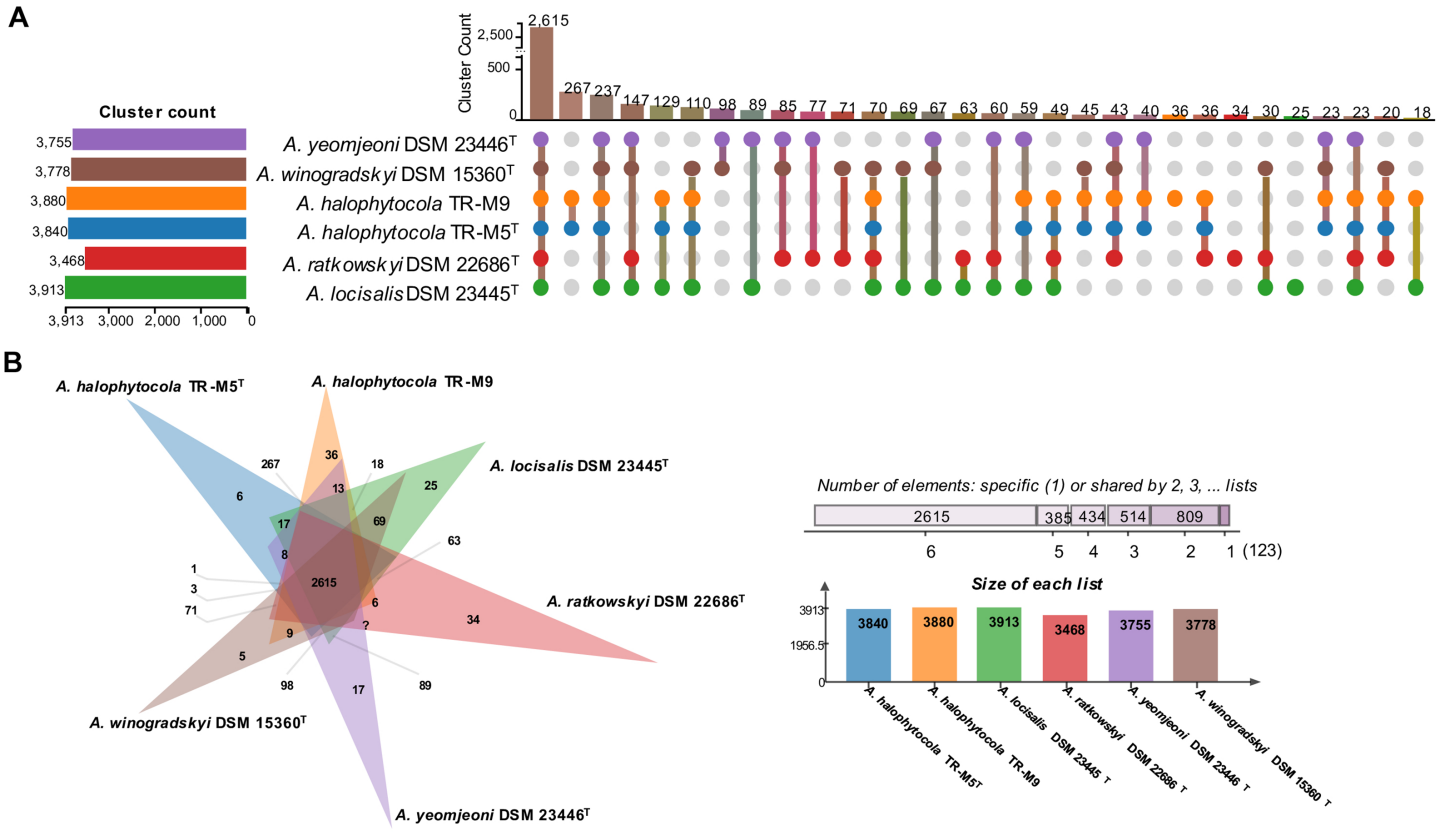
Distribution of shared orthologous gene clusters among selected strains. **(A)** The UpSet plot and **(B)** the Venn diagram showing the distribution of shared orthologous gene clusters among the selected strains. Strains: 1, TR-M5^T^; 2, TR-M9; 3, *A. locisalis* DSM 23445^T^; 4, *A. yeomjeoni* DSM 23446^T^; 5, *A. winogradskyi* DSM 15360^T^; 6, *A. ratkowskyi* DSM 22686^T^.

Orthologous cluster analysis revealed that all six strains shared 2,615 clusters. Additionally, TR-M5^T^ and TR-M9 possessed 267 unique clusters, while 147 clusters were unique to the other strains (Figure 3A). This finding indicates that TR-M5^T^ and TR-M9 have distinct genomic features that differentiate them from the other strains studied. Furthermore, TR-M5^T^ exhibited six additional unique clusters, and TR-M9 exhibited 36 additional unique clusters, highlighting further genomic differences between these two strains (Figure 3B).

**Figure 4:**
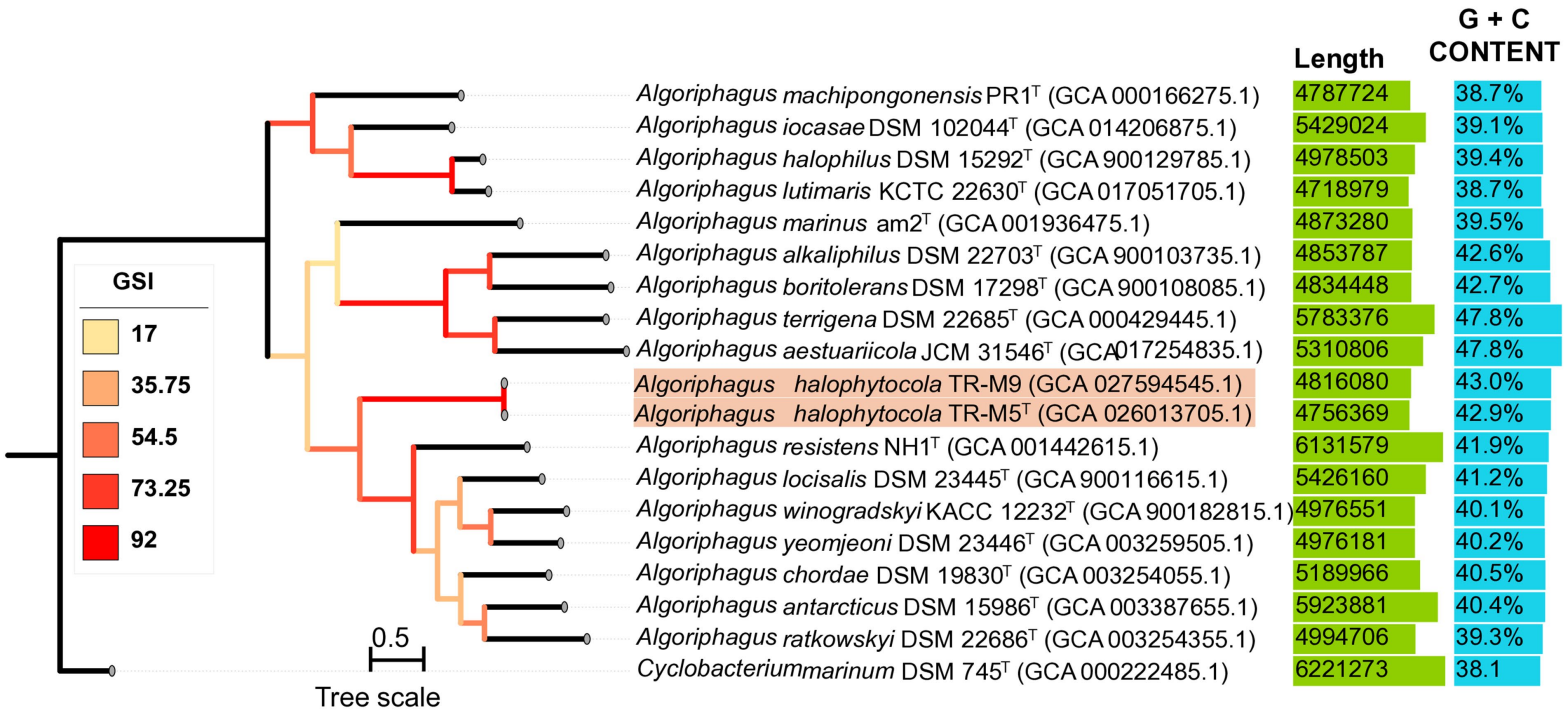
A phylogenomic tree analysis of strains TR-M5^T^ and TR-M9 using 92 core UBCGs. A phylogenomic tree was constructed using the maximum likelihood (ML) algorithm based on Up-to-date bacterial 92 core genes (UBCGs), which comprise a concatenated alignment of essential genes, including strains TR-M5^T^, TR-M9, and related type strains. Gene support indices are indicated at branching points. The 92-core gene is described by Na et al. (2018). The scale bar on the three represents 0.1 substitutions per nucleotide position.

The whole-genome phylogenetic tree based on 92 core gene sets provided additional support for the placement of strain TR-M5^T^ within the phylogenetic lineage of the genus *Algoriphagus* (Figure 4), consistent with the findings from the 16S rRNA gene-based phylogenetic analysis. This corroborates the evolutionary relatedness of strain TR-M5^T^ to other members of the genus *Algoriphagus*, emphasizing its distinct taxonomic position within this group.

### 3.4 Functional genomic analysis of plant growth promotion

Strain TR-M5^T^ was isolated from halophytes, and KEGG annotation analysis revealed several genomic features supporting its potential as an endophytic bacterium. One key feature is the presence of quorum sensing (QS) systems, particularly those involving N-acylhomoserine lactones (AHL), QS systems regulate various functions such as biofilm formation and symbiotic interactions, which are essential for successful endophytic colonization (Fray, 2002).

In addition, the genomic ability of TR-M5^T^ to synthesize phytohormones such as IAA through the tryptophan biosynthetic pathway suggests its potential to directly affect plant growth and development (Pantigoso et al., 2022; Supplementay Table S2). Similarly, the polyamine biosynthesis pathway in TR-M5^T^ enables the production of key polyamines, including putrescine, spermidine, and spermine, which are involved in regulating cell division, root growth, and stress responses. These polyamines also play a role in stabilizing cellular membranes and scavenging reactive oxygen species, thereby enhancing plant resilience under stress conditions(Alcázar et al., 2010; Supplementay Table S2). Furthermore, TR-M5^T^ can also synthesize and accumulate various protective compounds that help the host plant combat osmotic stress. Notably, the pathway for proline and betaine synthesis were identified, which are crucial for stress tolerance (Banu et al., 2009; Supplementay Table S2).

According to the dbCAN3 annotation, *A. ratkowskyi* DSM 22686^T^ displayed the highest count of carbon metabolism enzymes, totaling 402 annotated enzymes (Supplementay Figure S3). TR-M5^T^ with 239 enzymes, TR-M9 with 234 enzymes, *A. winogradskyi* DSM 15360^T^ had 218 enzymes, *A. locisalis* DSM 23445^T^ had 213 enzymes, and *A. yeomjeoni* DSM 23446^T^ had 212 enzymes. This variability in enzyme counts could be attributed to the diverse habitats of these bacteria. *A. ratkowskyi* DSM 22686^T^ originates from polar regions, while other bacteria, including TR-M5^T^, inhabit high-salt environments such as marine ecosystems.

All strains exhibited a high abundance of glycoside hydrolase (GH) family enzymes, constituting 39–46% of the total. Glycosyltransferase (GT) family enzymes were the second most prevalent, comprising 25–38%. Notably, TR-M5^T^, as a plant endophyte, showed a significantly elevated presence of GT family enzymes, particularly the GT2 family, compared to other marine-related reference bacteria. This highlights the potential importance of TR-M5^T^ in plant-microbe interactions and its unique enzymatic capabilities in carboxylate metabolism.

Overall, the genomic analysis of TR-M5^T^ suggests its promising role in promoting plant growth and aiding in stress tolerance, making it a valuable candidate for agriculture application, especially in saline environments.

### 3.5 Physiological characteristics

Strains TR-M5^T^ and TR-M9 were identified as Gram-stain-negative, aerobic, catalase-and oxidase-positive bacteria. They exhibited a non-motile, rod-shaped morphology with dimensions ranging from approximately 0.3–0.4 μm in width and 0.7–1.1 μm in length (Supplementay Figure S4). Colonies grown on MA for 3 days at 25°C displayed circular shapes, smooth textures, and a light pink color, measuring about 1 mm in diameter. Optimal growth conditions for both strains were determined to be at 25°C, pH 7, and a NaCl concentration of 6%. They demonstrated growth within a temperature range of 4–42°C, pH range of 6.0–9.0, and NaCl concentration range of 0–8.0% (Table 2).

**Table 2:** Differential physiological features comparison of TR-M5^T^, TR-M9, and other closely related type strains.

Characteristics	1	2	3^a^	4^b^	5^c^	6^d^
Cell size (μm)	0.3–0.4 × 0.7–1.1	0.3–0.4 × 0.7–1.1	0.4–0.7 × 1.5–3.0^a^	0.4–0.7 × 1.5–2.5^b^	0.5–0.7 × 2–10^c^	0.3–0.4 × 0.3–0.9^d^
Growth condition						
Temperature range (°C; optimum)	4–42 (25)	4–42 (25)	4–35 (30)^a^	4–35 (25–30)^b^	4–39 (25–28)^c^	−2–25 (16–19)^d^
NaCl tolerance (%, w/v; optimum)	0–8 (6)	0–8 (6)	0–9 (2)^a^	0–9 (2)^b^	0–6 (ND)^c^	0.6–5.8 (ND)^d^
pH range	6.0–9.0 (7)	6.0–9.0 (7)	5.5–8 (7–8)^a^	5–8 (7–8)^b^	ND^c^	6–8^d^
API 50CH						
D-Arabinose	−	−	+	−	−	w
L-Arabinose	−	−	w	−	−	−
D-Xylose	−	−	+	−	w	w
Galactose	−	−	+	−	w	−
D-Glucose	−	−	+	−	+	w
D-Fructose	−	−	+	−	w	−
D-mannose	−	w	+	−	+	+
Sorbitol	−	−	+	−	−	−
Methyl-D-mannoside	−	−	w	−	w	+
Methyl-D-glucoside	−	−	w	−	w	+
N-acetyl-glucosamine	−	−	−	−	−	+
Amygdalin	−	−	−	−	−	+
Arbutin	w	w	w	−	w	+
Salicin	−	−	w	−	w	+
Cellobiose	−	−	+	−	+	+
Maltose	−	−	+	−	+	+
Lactose	−	−	+	w	w	+
Melibiose	−	−	+	−	w	w
Sucrose	−	−	+	−	+	w
Trehalose	−	−	+	−	w	w
D-raffinose	−	−	w	−	w	−
Turanose	−	−	w	−	−	w
Lyxose	−	−	w	−	−	w
L-fucose	−	−	w	−	−	−
API 20NE						
Esculin hydrolysis	w	+	+	w	+	+
Paranitrophenyl-β-D-galactopyranoside	w	+	+	+	+	+
API ZYM						
Valine arylamidase	+	+	+	+	+	w
Cystine arylamidase	+	+	+	+	+	w
Trypsin	+	+	+	+	+	−
Acid phosphatase	+	+	+	+	+	w
N-acetyl-β-glucosaminidase	w	w	+	+	+	+

1, TR-M5^T^; 2, TR-M9; 3, *A. locisalis* KCTC 12310^T^; 4, *A. yeomjeoni* KCTC 12309^T^; 5, *A. winogradskyi* KACC 12232^T^; 6, *A. ratkowskyi* KCTC 92714^T^. All strains are rod-shaped and non-motile and positive for catalase and oxidase activity. All data are from this study unless otherwise indicated. +, positive; −, negative; w, weak.

a Yoon et al. (2005b).

b Yoon et al. (2005a).

c Nedashkovskaya et al. (2004).

d Bowman et al. (2003).

Both strains exhibited positive activity for various enzymes, including alkaline phosphatase, esterase, esterase lipase, leucine arylamidase, valine arylamidase, cystine arylamidase, trypsin, α-chymotrypsin, acid phosphatase, naphthol-AS-BI-phosphohydrolase, β-galactosidase, α-glucosidase, β-glucosidase, N-acetyl-β-glucosaminidase (weak), and α-mannosidase, as determined through the API ZYM test. In the API 20NE test, strain TR-M5^T^ showed weak positivity for esculin hydrolysis and paranitrophenyl-β-D-galactopyranoside, while TR-M9 displayed positive results for both tests. In the API 50CH test, both strains showed weak positivity for arbutin and positivity for esculin. Additionally, TR-M9 showed a weak positive for D-mannose. Further distinctive characteristics of strain TR-M5^T^ in comparison to closely related type strains are provided in Table 2.

### 3.6 Chemotaxonomy characteristics

The major polar lipids of strain TR-M5^T^ included Phosphatidylethanolamine (PE), phosphatidylcholine (PC), an unidentified phospholipid (PL), and an unidentified lipid (L1). In contrast, strain TR-M9 contained PE, PC, an unidentified PL, and four unidentified lipids (L1–L4; Supplementay Figure S5). Notably, MK-7 was identified as the predominant isoprenoid quinone in both strains, TR-M5^T^ and TR-M9, with a peak area ratio of approximately 95–96%, consistent with the profile observed in *A. ratkowskyi* (Bowman et al., 2003).

Analysis of the major fatty acids (>10%) revealed iso-C_15:0_ and summed feature 3 (comprising C_16:1_ *ω*6*c* and/or C_16:1_ *ω*7*c*) as the prominent constituents in strains TR-M5^T^ and TR-M9 (Table 3). These fatty acid profiles serve as distinguishing features for strains TR-M5^T^ and TR-M9 when compared to closely related type strains cultured under similar conditions. Consequently, these results suggest that strains TR-M5^T^ and TR-M9 represent a novel species within the *Algoriphagus* genus.

**Table 3:** Fatty acid compositions of TR-M5^T^, TR-M9, and other closely related type strains.

Fatty acids	1	2	3	4	5	6
Saturated						
C_16:0_	1.4	1.4	1.5	0.7	1.3	2.1
Unsaturated						
anteiso-C_11:0_	2.9	3.2	2.7	0.0	4.3	2.0
iso-C_14:0_	1.1	0.9	0.5	0.8	0.5	2.0
anteiso-C_15:0_	1.8	2.4	1.4	2.3	2.0	5.2
iso-C_15:0_	**12.5**	**15.3**	**15.9**	**24.6**	**18.6**	**24.6**
iso-C_15:0_ 3-OH	3.5	2.7	2.5	3.2	2.5	1.8
iso-C_15:1_ G	1.1	1.0	4.7	5.3	5.3	2.5
iso-C_16:0_	6.5	6.4	4.6	3.8	4.6	5.4
iso-C_16:0_ 3-OH	2.1	2.4	2.8	4.9	3.1	3.8
iso-C_16:1_ H	7.0	7.6	5.7	5.1	4.9	4.9
iso-C_17:0_ 3-OH	4.8	5.2	6.1	8.3	8.7	8.1
C_15:1_ *ω*6*c*	0.0	0.0	0.0	0.0	0.0	1.2
C_16:1_ *ω*5*c*	5.8	6.3	4.6	2.7	5.3	7.5
C_17:1_ *ω*6*c*	2.8	1.8	2.0	0.9	1.5	0.4
Hydroxy						
C_16:0_ 3-OH	5.5	4.1	1.9	1.1	1.7	1.7
Summed feature^*^						
3	**31.2**	**29.6**	**35.7**	**30.5**	**29.6**	**20.5**
4	2.2	1.7	1.2	0.0	1.3	1.2
9	4.4	4.6	4.2	4.4	4.2	2.7

All data presented were obtained in this study. Major components (>10%) are shown in bold. Fatty acids that represented <1% in all strains are not shown. 1, TR-M5^T^; 2, TR-M9; 3, *A. locisalis* KCTC 12310^T^; 4, *A. yeomjeoni* KCTC 12309^T^; 5, *A. winogradskyi* KACC 12232^T^; 6, *A. ratkowskyi* KCTC 92714^T^. All the data were obtained in the present study.

* Summed feature 3 contains C_16:1_ *ω*6*c* and/or C_16:1_ *ω*7*c*; summed feature 4 comprised iso-C_17:1_ and/or anteiso B, and summed feature 9 comprised iso-C_17:1_ ω9c and/or C_16:0_ 10-methyl.

### 3.7 Plant growth-promoting traits of strains TR-M5^T^ and TR-M9 under salt stress

**Figure 5:**
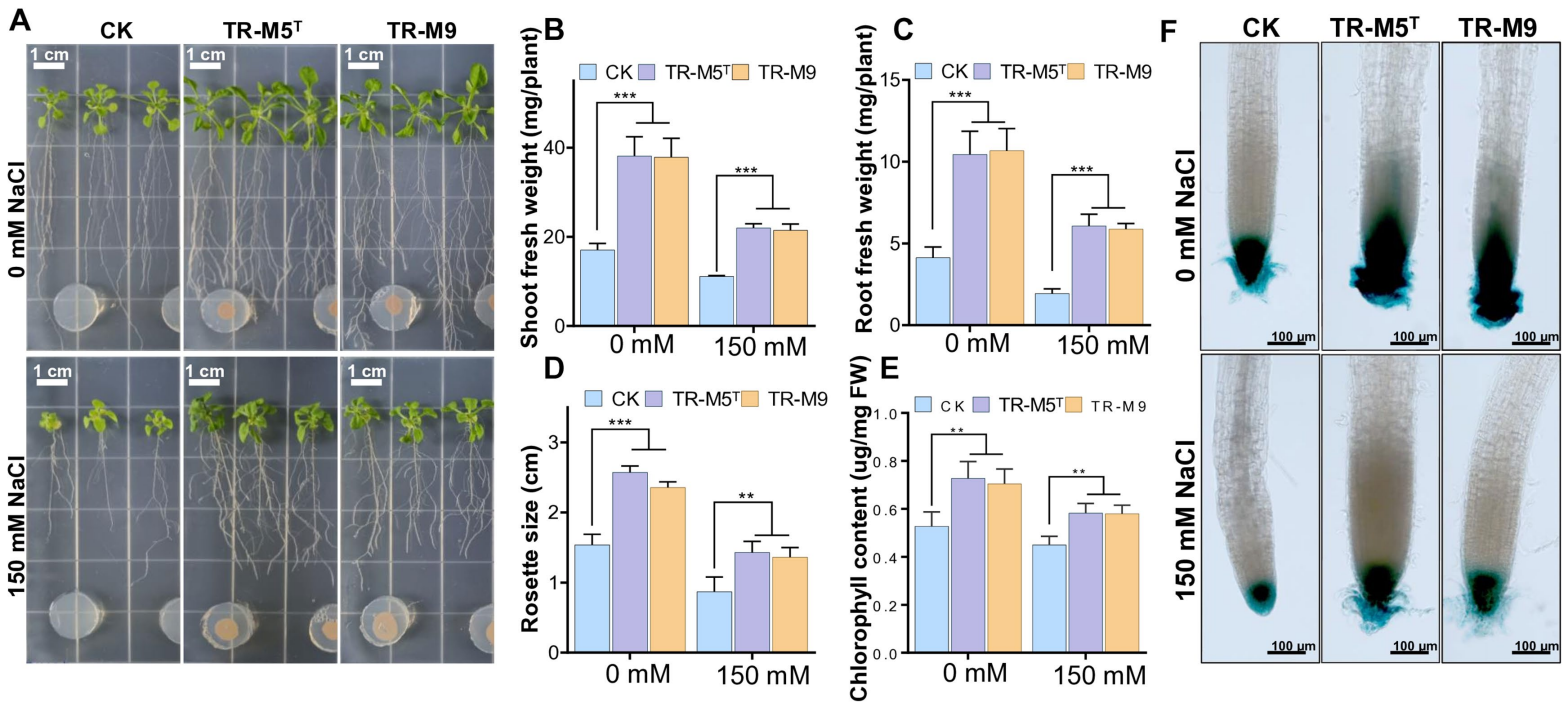
Growth of *Arabidopsis* seedlings under salt stress conditions. Seven-day-old *Arabidopsis* seedlings were co-cultivated with strains TR-M5^T^ and TR-M9 on 1/2 MS on salt conditions. **(A)** Morphological comparison of seedlings on salt conditions. **(B)** Shoot fresh weight. **(C)** Root fresh weight. **(D)** Rosette diameter. **(E)** Chlorophyll content. **(F)** DR5::GUS expression levels in root tips. Error bars indicate the standard deviation of the mean (*n* = 6). Asterisks represent significant differences between the co-culture and control (CK; Student’s *t*-test, **p ≤ 0.01, ***p ≤ 0.001). There was no significant difference observed between the TR-M5^T^ and TR-M9 treatment groups.

To evaluate the effects of TR-M5^T^ and TR-M9 on plant growth, we investigated their plant growth-promoting (PGP) traits under 150 mM NaCl salt concentrations. We measured growth parameters such as shoot fresh weight, root fresh weight, rosette diameter, and chlorophyll content. Increased sodium chloride concentration adversely affected plant health and growth (Figures 5A–E). However, compared to the CK plants, strains TR-M5^T^ and TR-M9 significantly restored rosette diameter, root fresh weight, shoot fresh weight, and chlorophyll content under saline conditions.

Specifically, under salt conditions, the shoot fresh weight of plants treated with TR-M5^T^ and TR-M9 was approximately 1.9 times greater compared to the CK (Figure 5B). The root fresh weight was about 3.0–3.1 times greater in treated plants compared to CK (Figure 5C). Additionally, the rosette size was approximately 1.6 times larger in treated plants compared to CK (Figure 5D). Moreover, plants cultured with TR-M5^T^ and TR-M9 exhibited significantly higher overall growth parameters, including chlorophyll content, which was 1.3 times greater than that of CK under salt conditions (Figure 5E).

Furthermore, the PGP effects of strains TR-M5^T^ and TR-M9 on primary roots and their relationship with auxin signaling were analyzed using transgenic *Arabidopsis* seedlings with the DR5::GUS promoter (Ulmasov et al., 1997). The treatment group, compared to the CK, significantly induced auxin production in the root tips under both salt and non-salt stress conditions, which also explains the increased number of secondary roots in the treatment group (Figure 5F).

Our results demonstrated that strains TR-M5^T^ and TR-M9 significantly enhanced the overall growth parameters of *Arabidopsis* seedlings under salt stress. This suggests that TR-M5^T^ and TR-M9 may function as plant probiotics, promoting growth under standard conditions and enhancing salinity resistance.

### 3.8 Estimation of plant growth-promoting traits of strains TR-M5^T^ and TR-M9

Strain TR-M5^T^ produced up to 52.4 μg/ml of IAA when supplemented with tryptophan, while stain TR-M9 produced 51.5 μg/ml of IAA under the same conditions (Supplementay Figure S6A). Additionally, the proline production of both TR-M5^T^ and TR-M9 ranged from 138 to 146 μg/ml (Supplementay Figure S6B), suggesting their potential role in enhancing plant stress tolerance. Proline is known for its protective effects against osmotic stress in plants (Liang et al., 2013). Both strains exhibited cellulase and gelatinase activities (Supplementay Figure S6C,D) but tested negative for amylase production. They could not produce siderophores or exhibit phosphate-solubilizing activity.

These characteristics suggest that stains TR-M5^T^ and TR-M9 possess multiple plant growth-promoting traits, making them promising candidates for enhancing plant growth and stress resilience in agricultural applications.

## 4 Discussion

The genus *Algoriphagus* has been previously identified in a variety of environments, but its role as a plant growth-promoting bacterium (PGPB) has not been well-documented. The isolation of TR-M5^T^ and TR-M9 from the halophyte *Salicornia europaea* highlights their adaptation to high-salinity environments, which is further supported by their ability to produce IAA and proline—key compounds involved in plant growth and stress response (Kang et al., 2020). IAA, a well-known phytohormone, is critical in regulating plant growth, cell division and lateral root development (Teale et al., 2006). The co-cultivation of *Arabidopsis* with strains TR-M5^T^ and TR-M9 demonstrated a significant enhancement in overall plant growth and lateral root development. GUS staining assays further indicated a marked increase in auxin production within the root tips of *Arabidopsis* seedlings following treatment with TR-M5^T^ and TR-M9.

Proline accumulation in plants is a well-documented response to osmotic stress, and the synthesis of this osmoprotectant by TR-M5^T^ and TR-M9 is particularly noteworthy. Proline not only stabilizes proteins and membranes but also acts as a free radical scavenger, reducing oxidative stress within plant cells (Singh et al., 2020; Altuntaş and Terzi, 2021). Additionally, the cellulase and gelatinase activities exhibited by these strains suggest they may assist in breaking down complex carbohydrates and proteins in the soil, potentially enhancing nutrient availability for plants. These combined traits underline their potential utility in agricultural settings where salinity and nutrient availability are limiting factors for crop productivity.

The genomic characteristics of TR-M5^T^ and TR-M9, including their unique orthologous gene clusters and functional annotations, set them apart from other *Algoriphagus* species. The presence of glycosyltransferase (GT) enzymes, particularly within the GT2 family, suggests a potential role in plant-microbe interactions, such as the modification of cell walls or the synthesis of extracellular polysaccharides (Chen et al., 2022). These activities could contribute to the bacteria’s ability to adapt to their environment and interact with plant hosts, although further studies are needed to confirm the specific functions of these enzymes. Additionally, the presence of quorum sensing (QS) systems in TR-M5^T^ hints at the possibility of regulating bacterial population density and coordinating activities within the plant host (Loera-Muro et al., 2021), but the exact impact on plant growth and health requires more detailed investigation.

### 4.1 Description of *Algoriphagus halophytocola* sp. nov

*Algoriphagus halophytocola* (ha.lo.phy.to'co.la. Gr. masc. n. *hals* salt; Gr. neut. n. *phyton*, plant; L. suff.—*cola* (from L. n. *incola*) inhabitant, dweller; N.L. masc. n. *halophytocola* inhabitant of a halophyte, *Salicornia europaea*).

Cells are Gram-negative, aerobic, non-motile, and rod-shaped that are 0.3 to 0.4 μm in wide and 0.7 to 1.1 μm in long. Colonies display a smooth, circular morphology with a light pink color and reach a diameter of approximately 1 mm after 3 days of incubation at 25°C. Cells grow at 4 to 42°C (with the optimum at 25°C), pH levels ranging from 6.0 to 9.0 (optimum pH 7.0), and NaCl concentrations from 0 to 8.0% (w/v; optimum 6.0%). The bacterium is catalase-and oxidase-positive and demonstrates growth on various media including MA, LB, and R2A. The predominant fatty acids identified are iso-C_15:0_ and summed feature 3 (C_16:1_ *ω*6*c* and/or C_16:1_ *ω*7*c*). The major polar lipids include phosphatidylethanolamine, phosphatidylcholine, an unidentified phospholipid, and an unidentified lipid, while MK-7 serves as the primary respiratory quinone. In the API 20NE kit test, the bacterium exhibits weak positive results for esculin hydrolysis and paranitrophenyl-β-D-galactopyranoside. In the API 50CH test, demonstrates weak positive results for arbutin and positive results for esculin. In the API ZYM test, positive results are observed for various enzymes including alkaline phosphatase, esterase, esterase lipase, leucine arylamidase, valine arylamidase, cystine arylamidase, trypsin, α-chymotrypsin, acid phosphatase, naphthol-AS-BI-phosphohydrolase, β-galactosidase, α-glucosidase, β-glucosidase, N-acetyl-β-glucosaminidase, and α-mannosidase.

The GenBank accession numbers for the 16S rRNA gene sequences of strain TR-M5^T^ and TR-M9 are OP720972 and OQ048311, respectively. Additionally, the whole-genome sequences are available under the accession numbers CP110226 and CP115160. Strains TR-M5^T^ and TR-M9 are available through the Korean Collection for Type Cultures (KCTC 92720^T^, KCTC 92721) and the Guangdong Microbial Culture Collection Center (GDMCC 1.3797^T^, GDMCC 1.3798).

## 5 Conclusion

This study highlights the potential of *Algoriphagus halophytocola* sp. nov. as a novel PGPB with unique adaptations to high-salinity environments. The ability of strains TR-M5^T^ and TR-M9 to produce IAA and proline, coupled with their cellulase and gelatinase activities, suggests that they can enhance nutrient availability and support plant growth under saline conditions. The genomic features of these strains, particularly their glycosyltransferase enzymes and quorum sensing systems, provide further insights into their potential roles in plant-microbe interactions. These findings indicate that *Algoriphagus halophytocola* could be a valuable bioinoculant for improving crop productivity in saline soils, contributing to the development of sustainable agricultural practices in challenging environments.

## Data Availability

The datasets presented in this study can be found in online repositories. The names of the repository/repositories and accession number(s) can be found in the article/Supplementary material.

## Footnotes

^1^https://lpsn.dsmz.de/genus/algoriphagus

^2^https://www.ncbi.nlm.nih.gov/genbank

^3^www.ezbiocloud.net

^4^www.ezbiocloud.net/tools/orthoani

^5^https://www.kegg.jp/blastkoala/
