# Supplementary material for: Improving plant salt tolerance through Algoriphagus halophytocola sp. nov., isolated from the halophyte Salicornia europaea
Source: Front Microbiol. 2024 Oct 21;15:1466733. doi: 10.3389/fmicb.2024.1466733 (PMC11532033; doi:10.3389/fmicb.2024.1466733)
Supplement: Supplementary file 1 [file Data_Sheet_1.PDF]

Supplementary materials

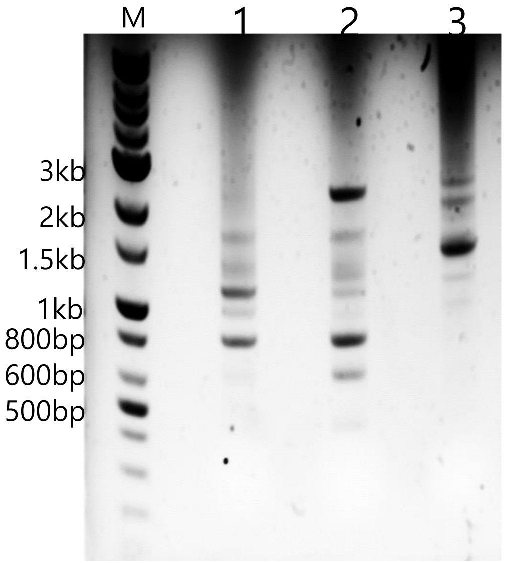

Figure S1. BOX-PCR fingerprint analysis. Strains: 1, TR-M5^T; 2, TR-M9; 3, *A. locisalis* KCTC 12310^T. M, molecular weight marker.

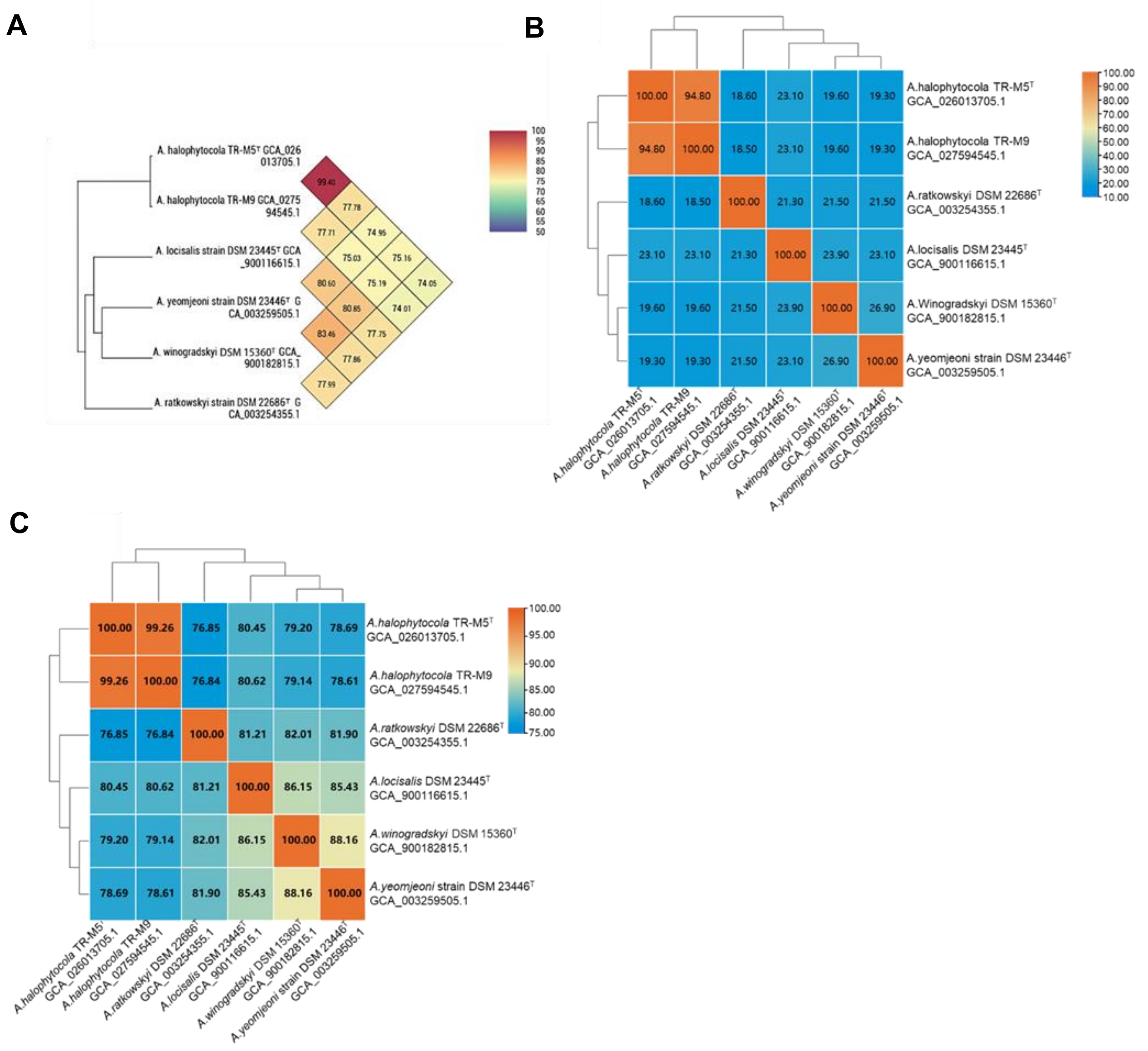

Figure S2. Matrix heatmap of overall genome relatedness indexes (ORGI). (A) Assessment of Average Nucleotide Identity (ANI) Values; (B) Digital DNA–DNA hybridization (dDDH) values; (C) Evaluation of Average Amino Acid Identity (AAI) Values.

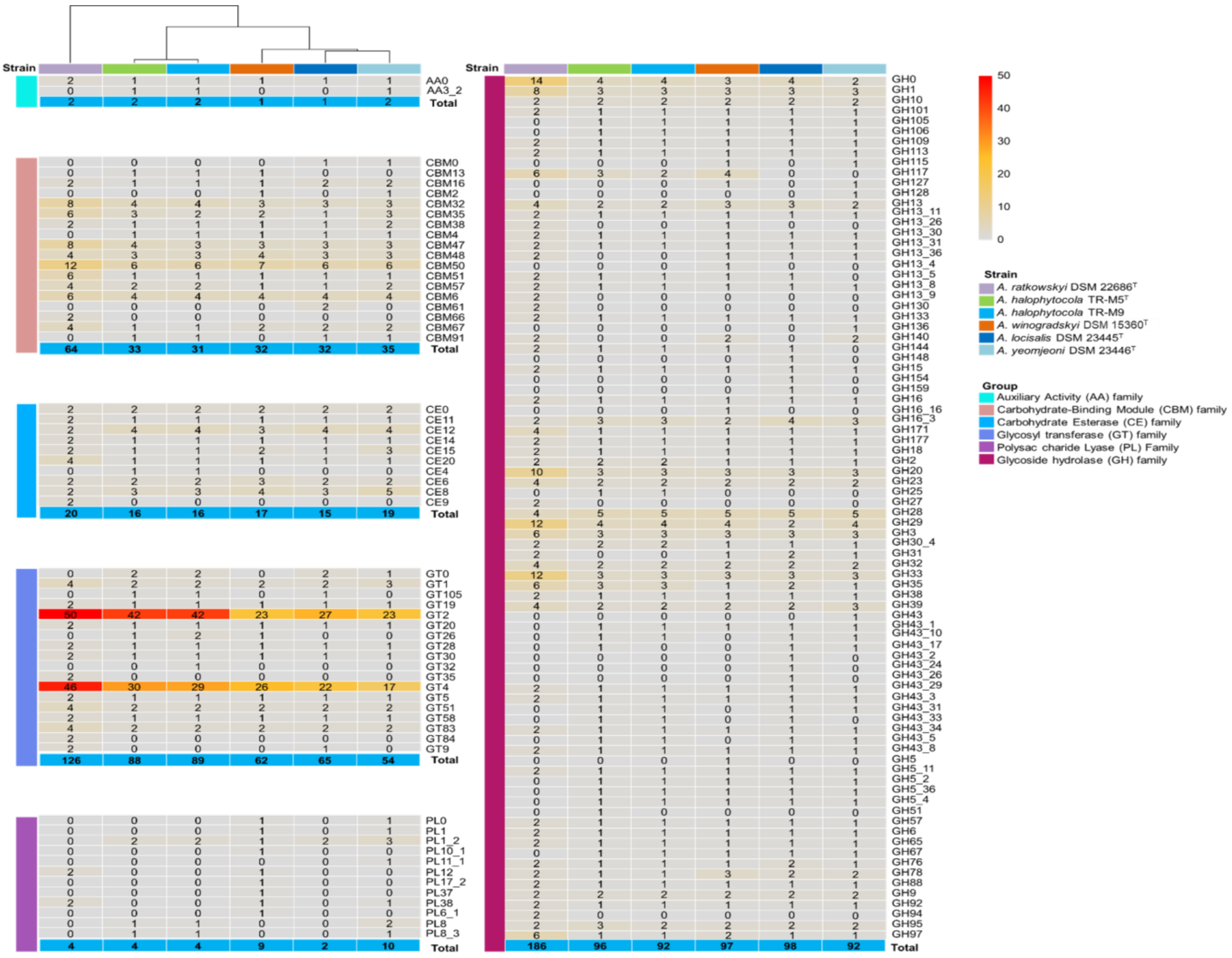

Figure S3. Carbohydrate-active enzyme (CAZy) families in TR-M5^{T}, TR-M9, and related type strains.

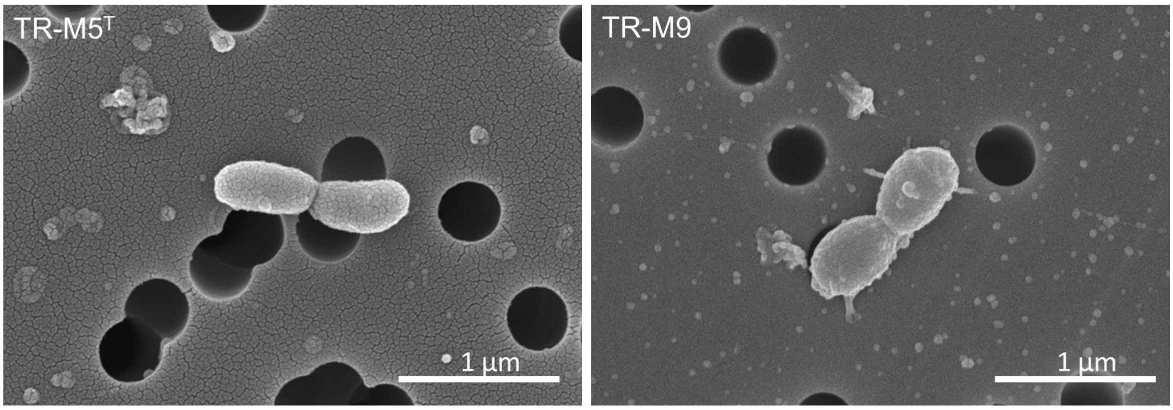

Figure S4. Scanning electron microscopy micrographs of the strains TR-M5^T and TR-M9. Scale bar, 1 µm.

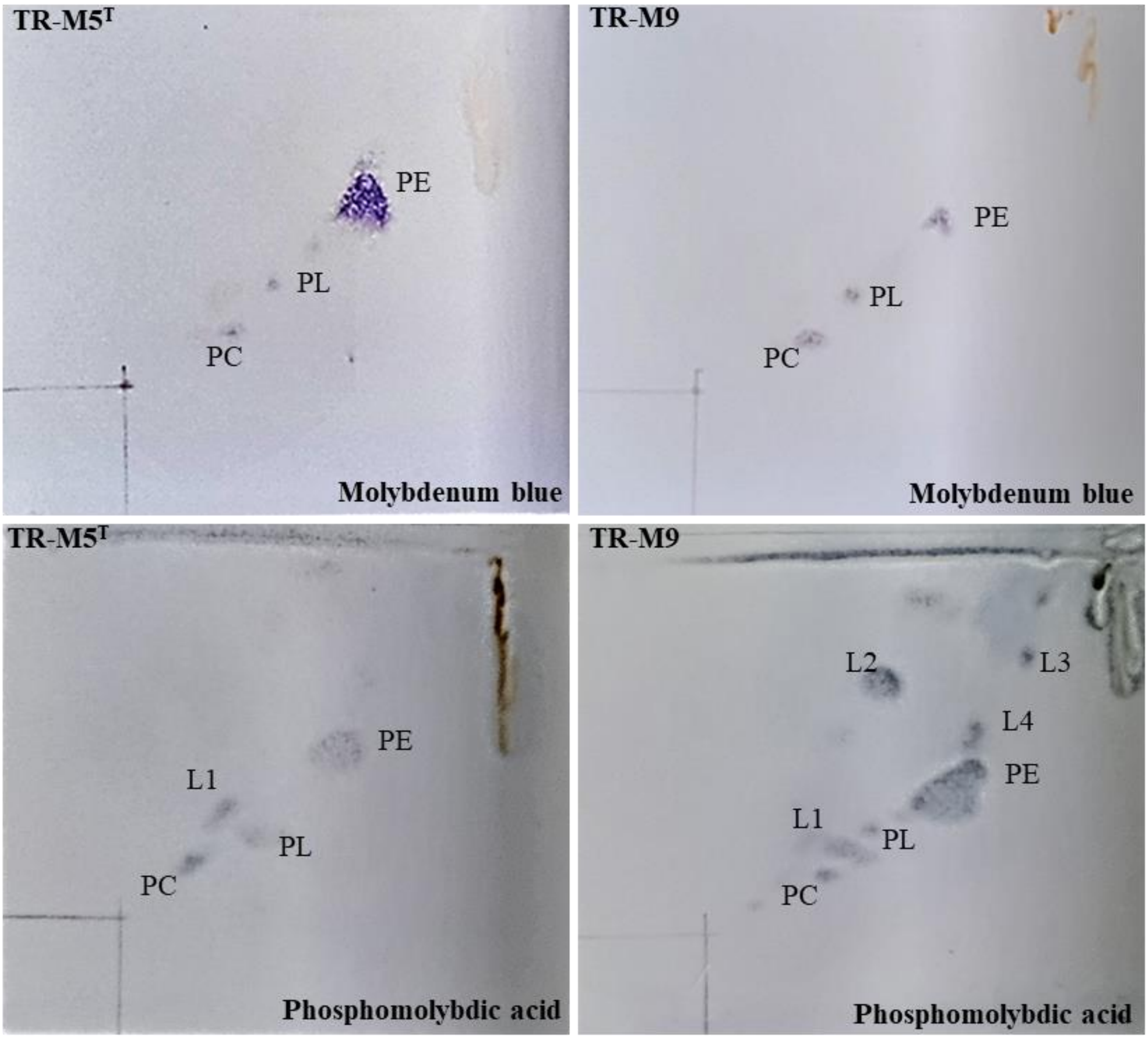

Figure S5. Two-dimensional TLC separation of polar lipids of strainTR-M5^{T} and TR-M9. The polar lipid profile was visualized using molybdenum blue for phospholipids, phosphomolybdic acid for total lipids.

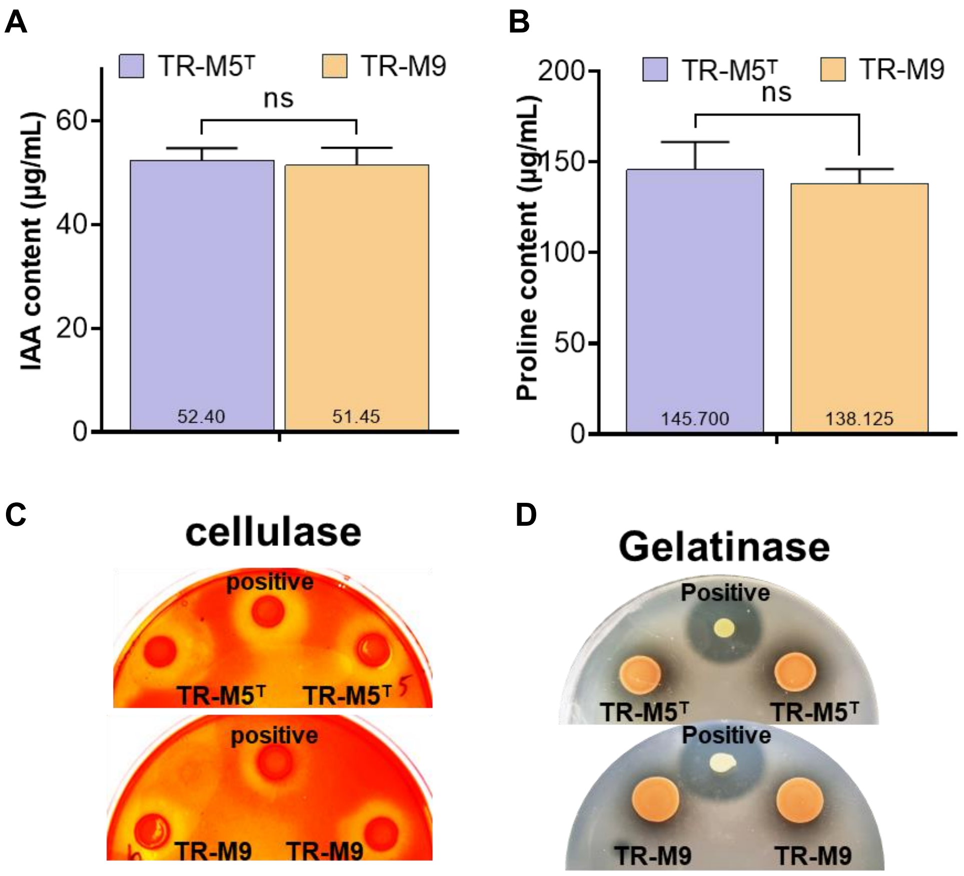

Figure S6. Biochemical activities of TR-M5^T and TR-M9. (A) IAA production, (B) Proline production, (C) Cellulase activity, (D) Gelatinase activity.

Table S1. Functional classification of proteins in the genome of strains TR-M5^T and TR-M9 based on Clusters on Orthologous Groups (COG) and biosynthesis gene cluster distribution.

	eggNOG Function Description	TR-M5^T		TR-M9	
		**count**	**%**	**count**	**%**
	**Total eggNOG Function**	**3781**	**100**	**3833**	**100**
	**Information storage and processing**				
J	Translation, ribosomal structure, and biogenesis	153	4.05	153	3.99
A	RNA processing and modification	0	0	0	0
K	Transcription	142	3.76	139	3.63
L	Replication, recombination, and repair	135	3.57	146	3.81
B	Chromatin structure and dynamics	1	0.03	1	0.03
	**Total**	**431**	**11.41**	**439**	**11.46**
	**Cellular processes and signaling**				
D	Cell cycle control, cell division, chromosome partitioning	17	0.45	18	0.47
Y	Nuclear structure	0	0	0	0
V	Defense mechanisms	68	1.8	65	1.7
T	Signal transduction mechanisms	161	4.26	166	4.33
M	Cell wall/membrane/envelope biogenesis	250	6.61	255	6.65
N	Cell motility	1	0.03	1	0.03
Z	Cytoskeleton	0	0	2	0.05
U	Intracellular trafficking, secretion, and vesicular transport	25	0.66	25	0.65
O	Posttranslational modification, protein turnover, chaperones	113	2.99	109	2.84
	**Total**	**635**	**16.8**	**641**	**16.72**
	**Metabolism**				
C	Energy production and conversion	136	3.6	136	3.55
G	Carbohydrate transport and metabolism	161	4.26	164	4.28
E	Amino acid transport and metabolism	237	6.27	233	6.08
F	Nucleotide transport and metabolism	55	1.45	57	1.49
H	Coenzyme transport and metabolism	81	2.14	81	2.11
I	Lipid transport and metabolism	82	2.17	82	2.14
P	Inorganic ion transport and metabolism	196	5.18	190	4.96
Q	Secondary metabolites biosynthesis, transport and catabolism	34	0.9	34	0.89
	**Total**	**982**	**25.97**	**977**	**25.50**
	**Poorly characterized**				
R	General function prediction only	250	6.61	253	6.60
S	Function unknown	1483	39.22	1523	39.73
	**Total**	**1733**	**45.83**	**1776**	**46.33**

Table S2. Functional categories of genes involved in plant growth promotion and salinity stress tolerance mechanisms.

Classification		KO Numbers	Gene	Name	EC number
**Plant growth promotion**	**IAA biosynthesis**	K00766	*trpD*	Anthranilate phosphoribosyltransferase	[EC:2.4.2.18]
		K01609	*trpC*	Indole-3-glycerol phosphate synthase	[EC:4.1.1.48]
		K01657	*trpE*	Anthranilate synthase component I	[EC:4.1.3.27]
		K01658	*trpG*	Anthranilate synthase component II	[EC:4.1.3.27]
		K01695	*trpA*	Tryptophan synthase alpha chain	[EC:4.2.1.20]
		K01696	*trpB*	Tryptophan synthase beta chain	[EC:4.2.1.20]
		K01817	*trpF*	Phosphoribosylanthranilate isomerase	[EC:5.3.1.24]
		K13497	*trpGD*	Anthranilate Synthase/phosphoribosyltransferase	[EC:4.1.3.27 2.4.2.18]
	**Polyamine biosynthesis**	K01585	*speA*	Arginine decarboxylase	[EC:4.1.1.19]
		K01480	*speB*	Agmatinase	[EC:3.5.3.11]
		K10536	*aguA*	Agmatine deiminase	[EC:3.5.3.12]
		K12251	*aguB*	N-carbamoylputrescine amidase	[EC:3.5.1.53]
		K01476	*rocF*	Arginase	[EC:3.5.3.1]
**Salinity stress tolerance**	**Proline biosynthesis**	K00147	*proA*	Glutamate-5-semialdehyde dehydrogenase	[EC:1.2.1.41]
		K00931	*proB*	Glutamate 5-kinase	[EC:2.7.2.11]
		K00286	*proC*	Pyrroline-5-carboxylate reductase	[EC:1.5.1.2]
		K13821	*putA*	RHH-type transcriptional regulator, Proline utilization regulon repressor / Proline dehydrogenase / delta 1-pyrroline-5-carboxylate dehydrogenase	[EC:1.5.5.21.2.1.88]
	**Betaine biosynthesis**	K00108	*betA*	Choline dehydrogenase	[EC:1.1.99.1]
